# Intersectin: The Crossroad between Vesicle Exocytosis and Endocytosis

**DOI:** 10.3389/fendo.2013.00109

**Published:** 2013-08-27

**Authors:** Olga Gubar, Dmytro Morderer, Lyudmila Tsyba, Pauline Croisé, Sébastien Houy, Stéphane Ory, Stéphane Gasman, Alla Rynditch

**Affiliations:** ^1^State Key Laboratory of Molecular and Cellular Biology, Institute of Molecular Biology and Genetics, Kyiv, Ukraine; ^2^Department of Functional Genomics, Institute of Molecular Biology and Genetics, Kyiv, Ukraine; ^3^Institut des Neurosciences Cellulaires et Intégratives, Centre National de la Recherche Scientifique-Unité Propre de Recherche 3212, Université de Strasbourg, Strasbourg, France

**Keywords:** intersectin, exocytosis, endocytosis, neurons, neuroendocrine cells, Cdc42, scaffold

## Abstract

Intersectins (ITSNs) are a family of highly conserved proteins with orthologs from nematodes to mammals. In vertebrates, ITSNs are encoded by two genes (*itsn1* and *itsn2*), which act as scaffolds that were initially discovered as proteins involved in endocytosis. Further investigation demonstrated that ITSN1 is also implicated in several other processes including regulated exocytosis, thereby suggesting a role for ITSN1 in the coupling between exocytosis and endocytosis in excitatory cells. Despite a high degree of conservation amongst orthologs, ITSN function is not so well preserved as they have acquired new properties during evolution. In this review, we will discuss the role of ITSN1 and its orthologs in exo- and endocytosis, in particular in neurons and neuroendocrine cells.

## ITSN Family of Scaffold Proteins

Intersectins (ITSNs) are multifunctional scaffold proteins implicated in several cellular mechanisms, including membrane trafficking (clathrin- and caveolin-mediated endocytosis, secretagogue-evoked exocytosis) and receptor-dependent signaling (Ras-MAPK and Rho GTPase regulation, EGF receptor ubiquitylation) to name a few [extensively reviewed in (1–3)]. Based on the high involvement of ITSNs in membrane trafficking, this mini review will focus on the role of these proteins in exocytosis and endocytosis in neurosecretory cells such as neurons and neuroendocrine cells, and will discuss how ITSNs could be key players in coupling exocytosis to endocytosis.

**Figure 1:**
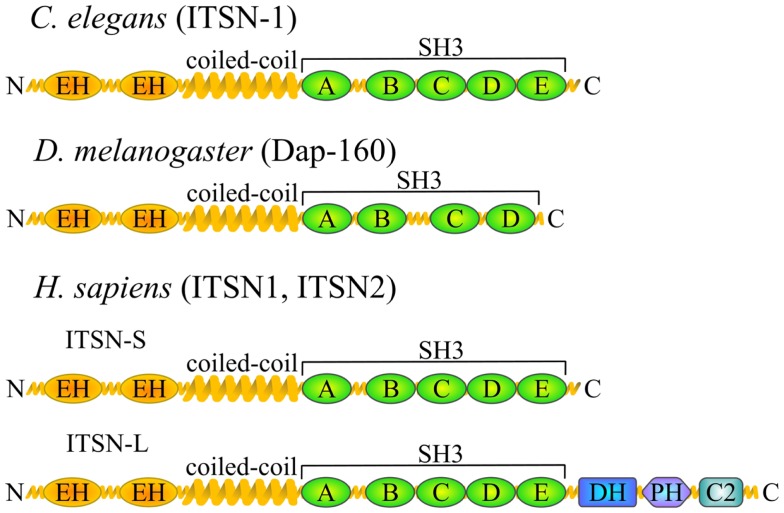
**ITSN orthologs and domain composition**. Schematic representation of the ITSN proteins in different species depicting the position of various functional domains. EH, Eps15 homology domain; SH3, Src homology 3 domain; DH, Dbl homology domain; PH, pleckstrin homology domain; C2, Ca^2+^-binding domain. Vertebrates usually have two ITSN genes, both of which give rise to two major isoforms.

Intersectin is highly conserved in all metazoans examined so far and ITSN orthologs have been found in nematodes (*Caenorhabditis elegans*) (4), arthropods (*Drosophila melanogaster* Dap160 – Dynamin-associated protein 160 kDa) (5), fish (*Danio rerio*) (6), amphibians (*Xenopus laevis*) (7), and mammals (*Mus musculus*, *Rattus norvegicus*, *Homo sapiens*) (8–10) (Figure 1). ITSN is encoded by one gene in invertebrates. Its molecular organization consists of two N-terminal EH (Eps15 homology) domains followed by a coiled-coil region and four or five SH3 (Src homology 3) domains (5). In vertebrates, two genes encode ITSN proteins (ITSN1 and ITSN2). In addition, ITSNs exist in two main isoforms generated by alternative splicing: a short form (ITSN-S) that harbors the same domain organization as invertebrate ITSN and is ubiquitously expressed and a long form (ITSN-L) which has three additional domains in its C-terminal part [tandem of Dbl (DH) and pleckstrin homology (PH) domains and a C2 domain] (10). This extension has guanine nucleotide exchange factor (GEF) properties for Cdc42 (11), a small GTPase of the Rho family. The ITSN1-L is enriched in neurons (10, 12).

## ITSN1 in Neuronal Endocytosis

Multimodular ITSN1 has been shown to interact with numerous endocytic proteins, including dynamin, AP2 (adaptor protein 2), proteins from the Epsin family, and the synaptojanin phosphatase; its function in the control of endocytosis has been described in different cell types and organisms [reviewed in (1, 2)]. The role of ITSN1 in endocytosis in neurons was further demonstrated in functional assays performed in various model organisms. *C. elegans* containing deletions in the *itsn-1* gene were hypersensitive to the acetylcholine esterase inhibitor aldicarb that is widely used for unmasking neurotransmission defects. It causes rapid hypercontraction and eventual death of wild-type worms. This *itsn-1-*null aldicarb-hypersensitive phenotype could be rescued by expressing ITSN-1 in neurons, but not in muscles, indicating a pre-synaptic dysfunction in mutant animals (4). Another study revealed a decrease in the number of vesicles in neuromuscular junctions (NMJ) of *itsn-1*-null worms, implying that ITSN-1 was required for synaptic vesicle (SV) recycling (13). In both cases, the mutant worms were viable and exhibited no changes in growth and locomotor activity, implying that the nervous system functioned normally. This suggests that ITSN-1 is not essential for neuronal endocytosis in nematodes, but rather plays a regulatory role increasing the efficiency of this process. Worm ITSN-1 has been shown to form a complex with the Eps15 ortholog EHS-1 (13) and a mutation in the *ehs-1* gene also leads to SV depletion. However, *ehs-1*/*itsn-1* double mutants develop a phenotype similar to that of single mutants, supporting the idea that these proteins act in the same process (13, 14). Intriguingly, *itsn-1* and *ehs-1* mutants have opposite sensitivities to aldicarb, indicating that they have different effects on cholinergic neurotransmission, most likely at the pre-synaptic level (4). Finally, in addition to pre-synaptic functions, ITSN-1 is required for internalization of the GLR-1 (glutamate receptor 1) subunit of the α-amino-3-hydroxy-5-methyl-4-isoxazolepropionic acid-type glutamate (AMPA) receptors in the post-synaptic terminals of nematode command interneurons (15). Thus ITSN-1 seems to be important for both pre- and post-synaptic endocytosis in worms.

In contrast to nematodes, the loss-of-function mutation of the *ITSN* ortholog *Dap160* in *Drosophila* is lethal at late larval stages. Functional studies on the NMJ of mutant flies did not reveal significant changes in stimuli-evoked SV exocytosis; however, prolonged stimulation led to a severe decline in the excitatory post-synaptic potential (EPSP), indicating impairment of compensatory endocytosis (16, 17). As in nematodes, the number of vesicles in the active zones decreased in the NMJ of mutant *Drosophila* (16). In agreement with a function of ITSN in tethering endocytic proteins to the sites of endocytosis, the loss of Dap160 leads to mislocalization of dynamin, endophilin, and synaptojanin, resulting in decreased levels of these proteins in periactive synaptic zones (16, 17). Interestingly, overexpression of Dap160 also leads to a decrease in the level of synaptojanin, a 5′-phosphoinositide phosphatase involved in late stages of endocytosis, in NMJ synaptic terminals (18), indicating that Dap160/ITSN1 functions as a scaffold in endocytosis and is required to maintain endocytic proteins in synaptic terminals at levels necessary for efficient retrieval of SVs.

Studies on lamprey giant reticulospinal synapse have confirmed the data obtained in invertebrates concerning the involvement of ITSN1 in SV endocytosis. Perturbation of ITSN1 function by injecting either anti-ITSN1 antibodies or the SH3C domain into the pre-synaptic terminal resulted in an accumulation of clathrin-coated pits and a reduction in SV number, suggesting a defect in compensatory endocytosis (19, 20). It has also been shown that ITSN1 binds the AP2 clathrin adaptor both in lamprey and mammalian brain, suggesting that ITSN1 participates in the early steps of clathrin-mediated SV endocytosis. Moreover, such an interaction prevents ITSN1 from associating with synaptojanin 1 (20). These findings imply that ITSN1 temporally regulates SV endocytosis.

The data regarding the role of ITSN1 in SV endocytosis in mammals remain contradictory. ITSN1 along with Eps15 have been shown to form a complex with FCHo (Fer/Cip4 homology domain-only) proteins, that promotes the initiation of clathrin-coated pit formation – the step required for clathrin-mediated endocytosis of different cargo in various cell types, including SV endocytosis in neurons (21). ITSN1 isoforms have also been identified as members of the synaptotagmin I-associated endocytic complex in the synaptosomal fraction of rat brain, and were subsequently shown to co-localize with other members of this complex in the pre-synaptic transmitter-release face of the giant calyx-type synapse of the chick ciliary ganglion (22). Knockout of *ITSN1* in mice slows down SV endocytosis in neurons, although the animals are viable (23). However, another group found that SV endocytosis remains normal following ITSN1 knock-down in rat primary hippocampal neurons. This was demonstrated by the absence of a decrease in the uptake of FM4-64 membrane dye after KCl stimulation (24). Moreover, using capacitance measurements of plasma membrane in knockout mouse calyx of Held synapse, Sakaba and co-workers have recently shown that ITSN1 is not essential for endocytic SV recovery after pulse stimulation (25). Furthermore, several authors have reported that ITSN1 is mostly localized post-synaptically in hippocampal neurons, (24, 26), although some data indicating a pre-synaptic localization in mammalian synapses also exist (20, 25). Finally, recent electrophysiological studies did not reveal abnormalities in synaptic transmission either in ITSN1 knockout mice or in ITSN1/ITSN2 double knockout mice using a wide range of stimulation protocols, indicating that under the conditions tested SV recycling is not perturbed (27). Therefore it seems possible that in mammals, in contrast to invertebrates, ITSN1 acts predominantly post-synaptically; however, we cannot totally exclude it is an accessory protein in SV recycling.

Thus the role of ITSN in neuronal endocytosis apparently has not been conserved during evolution. In some groups of organisms (e.g., arthropods) ITSN is indispensable for normal SV recycling, whereas in higher evolved organisms it has become non-essential probably due to the development of alternative or compensatory mechanisms. In lower organisms, these functions are fulfilled by the short isoform, whereas in higher organisms (e.g., vertebrates) ITSN1-L is the likely candidate as it is the main isoform in neurons.

## ITSN1 Function in Exocytosis in Neuroendocrine Cells

There is much less evidence about the implication of ITSN in exocytosis. One of the first studies reported the interaction of ITSN1 with t-SNAREs [target Soluble NSF Attachment Protein (SNAP) Receptor] SNAP-23 and SNAP-25, but no functional consequence was demonstrated (9). A new specific function of ITSN1-L as GEF for Cdc42 in exocytosis was described in neuroendocrine cells. In regulated exocytosis, remodeling of the dense actin cortical network is an important step that is controlled by small GTPases. The key players in this process are the Rho family GTPases [reviewed in (28, 29)]. In the PC12 rat pheochromocytoma secretory model, Cdc42 was shown to be activated near the plasma membrane during exocytosis, where it recruits Neural Wiskott–Aldrich syndrome protein (N-WASP) and induces actin polymerization (30). ITSN1-L appeared to be an ideal candidate for Cdc42 activation at docking sites for secretory granules, because it is a specific GEF for Cdc42 and at the same time binds to its effector N-WASP (11). This leads to local polymerization of actin, thereby facilitating exocytosis. ITSN1-L was observed to co-localize with exocytic sites in PC12 and primary bovine chromaffin cells (31). Moreover, silencing of ITSN1 (as well as of Cdc42) significantly inhibits regulated exocytosis in PC12 cells, whereas overexpression of the C-terminal part of ITSN1-L (DH-PH-C2 domains) promotes exocytosis and peripheral actin polymerization in neuroendocrine cells (31, 32). These results were confirmed in *ITSN1*-null mice where exocytosis is also reduced in chromaffin cells (23). Finally, ITSN1 was very recently shown to regulate the replenishment of the fast-releasing SV pool in mouse calyx of Held synapse, possibly together with dynamin 1 and as a GEF for Cdc42 (25). Thus ITSN1-L displays novel properties in membrane trafficking distinct from those of ITSN1-S that were probably acquired during evolution.

## ITSN as a Plausible Link between Exo- and Endocytosis

Since ITSN1, especially the isoform ITSN1-L, is strongly implicated in both endo- and exocytosis it is an ideal protein for the coupling of these processes. Regulated exocytosis is always followed by compensatory endocytosis, which is also Ca^2+^-dependent and is stimulated together with exocytosis (33). Thus the presence of a calcium-binding C2 domain in ITSN-L may be a key for its implication in both exo- and endocytosis. Moreover, in studies on the chick ciliary ganglion ITSN1-S and ITSN1-L have been reported to be associated with the CaV2.2 calcium channels at transmitter-release sites (34).

In neurons, the active zone is highly structured to provide maximum secretory efficiency. The protein matrix, which consists of the cytoskeleton and scaffold proteins, ensures efficient SV docking, whereas electron-dense projections from this matrix serve to tether and maintain the ready-to-use SV pool (35). It is surrounded by a periactive zone that serves for SV recycling by endocytosis. A tight connection and coordination of SV release and recycling is provided by scaffolds [reviewed in (36)]. ITSN1 is a perfect candidate to fulfill this role. During synaptic activity, Dap160 has recently been shown to shuttle between active and periactive zones, ensuring the delivery of dynamin to the periactive zone for efficient bulk membrane retrieval (37). In the lamprey giant reticulospinal synapse, ITSN1 is also localized in the active zone of resting synapses, but re-localizes with its major binding partner dynamin to the periactive zone upon stimulation (19). These results support the idea that ITSN1 acts as a scaffold coupling vesicle fusion and internalization events.

**Figure 2:**
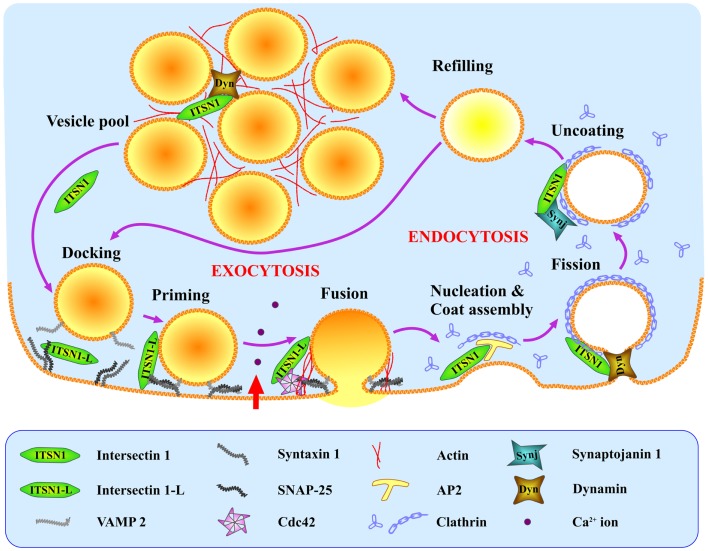
**Hypothetical model of ITSN1 coupling exo- and endocytosis in neuroendocrine cells**. SNAP-25 recruits ITSN1-L to the SNARE complex (VAMP2, SNAP-25, syntaxin 1) at the exocytic sites where ITSN1-L facilitates regulated exocytosis by activating Cdc42. Immediately after vesicle fusion and hormone release, ITSN1 promotes clathrin-mediated endocytosis of the granule membrane via AP2, and facilitates later stages of endocytosis together with dynamin and synaptojanin. ITSN1 then stays in the vesicle cluster (probably with dynamin) to be recruited back to the membrane for the next release cycle. ITSN1, total ITSN1(both short and long isoforms); ITSN1-L, long isoform of ITSN1.

Although to date there is currently no direct evidence that ITSN1 plays a role in compensatory endocytosis in neuroendocrine cells, such data do exist for other cell types (including neurons). Based on these data and the presence of ITSN1 partners in neuroendocrine cells, we propose the following hypothetical model of ITSN1 functioning in exo- and endocytosis in these cells (Figure 2). ITSN1 may function in both the release and retrieval of the dense-core granules. SNAP-25 could recruit ITSN1-L to exocytic sites where the latter activates Cdc42 thereby inducing actin polymerization and facilitating the late stages of secretion (9, 31, 32). After release, ITSN1-L remaining on the vesicle membrane could directly promote assembly of the endocytic complex, because the vesicle membrane has been shown to be preserved as an entity during the compensatory endocytosis in neuroendocrine cells (38). Otherwise, ITSN1 could be recruited *de novo* and induce clathrin coat assembly via AP2 (20). Then ITSN1, in turn, can recruit dynamin to provoke vesicle scission (8, 9). After vesicle dissociation from the membrane, AP2 could be replaced by synaptojanin 1, which takes part in the uncoating and dephosphorylation of PIP_2_ (phosphatidylinositol 4,5-bisphosphate) (20). The vesicle is then refilled and can be tethered ready for another cycle of exocytosis or added to the releasable vesicle pool. ITSN1 may accompany the vesicle throughout this cycle and remain in the releasable pool as it does in neurons (19). Thus ITSN1 could be a multipurpose player providing the crossroad between exo- and endocytosis in neurosecretory cells and an important regulator of these processes.

## Conflict of Interest Statement

The authors declare that the research was conducted in the absence of any commercial or financial relationships that could be construed as a potential conflict of interest.
